# Cell-type–specific repression reveals MC4R neurons as a site of action for ciliary obesity genes

**DOI:** 10.1016/j.molmet.2026.102454

**Published:** 2026-10-06

**Authors:** Abbey Diener Blake, Zhixuan Zhang, Coralie Alexandre, Anne Gorayeb, Mrinaalini Jain, Qi Liu, Rithu Pattali, Alicja Bednarska, Parleen Kaur Pandher, Sumei Zhang, Xinyu Yue, James Nuñez, Christian Vaisse

**Affiliations:** 1 Diabetes Center, University of California, San Francisco, San Francisco, CA, USA; 2 Department of Nutritional Sciences and Toxicology, University of California, Berkeley, Berkeley, CA, USA; 3 Department of Molecular and Cell Biology, University of California, Berkeley, Berkeley, CA, USA; 4 Chan Zuckerberg Biohub, San Francisco, San Francisco, CA, USA

**Keywords:** CRISPR interference (CRISPRi), MC4R neurons, Primary cilia, ADCY3, Obesity, Energy balance

## Abstract

**Objectives:**

Understanding how obesity-associated genes function within defined neuronal subpopulations remains a major barrier to translating human genetics into pathophysiological mechanisms. Melanocortin-4 receptor–expressing (MC4R) neurons are key regulators of energy homeostasis and represent a potential site of action for these genes. Here, we investigated whether obesity-associated ciliary genes regulate body weight through MC4R neurons *in vivo*, using a Cre-dependent CRISPR interference (CRISPRi) strategy that enables temporal, spatial, and cell-type–specific gene repression without permanent DNA modification.

**Methods:**

Gene repression was achieved in MC4R neurons using a Cre-dependent CRISPRi system with AAV-delivered sgRNAs, and physiological outcomes, including body weight and body composition, were assessed following gene downregulation.

**Results:**

CRISPRi-mediated repression of *Mc4r* in adult PVN^MC4R^ neurons reduced transcript and protein levels and induced progressive obesity, validating the approach. Downregulation of the ciliary adenylyl cyclase *Adcy3* specifically in adult PVN^MC4R^ neurons was sufficient to drive weight gain, establishing a cell-type–specific requirement for ADCY3 in body-weight regulation. An *in vivo* CRISPRi screen of obesity-associated ciliopathy genes *(Bbs10, Inpp5e, Ankrd26*) demonstrated that their repression in MC4R neurons is sufficient to promote obesity, identifying these neurons as a convergent site linking human ciliopathies to obesity.

**Conclusions:**

These findings define MC4R neurons as a critical neuronal population through which multiple obesity-associated ciliary genes act and establish a direct link between ciliary function in defined neuronal populations and organismal energy balance. This work provides insight into the neural mechanisms underlying obesity and a framework for linking human genetic variations to specific neuronal populations *in vivo*.

## Highlights

• Cre-dependent CRISPRi with AAV-delivered sgRNAs enables temporal, spatial, and cell-type-specific gene repression *in vivo.*
• ADCY3 is required for normal body weight regulation in adult PVN^MC4R^ neurons.
• Repression of *Bbs10*, *Inpp5e*, or *Ankrd26* in MC4R neurons is sufficient to promote obesity.
• MC4R neurons are a convergent site of action for primary cilia genes regulating body weight.

## 1 Introduction

Human genetic research has identified many genes that cause monogenic or syndromic obesity when mutated [[1], [2], [3], [4], [5], [6], [7], [8], [9], [10]], as well as hundreds of coding and noncoding variants associated with body weight [11,12]. Many of these genes act primarily in the brain, and the enrichment of genes near obesity-associated variants in the central nervous system (CNS) [13] suggests that additional genes will be found to function in CNS control of energy balance. Among these, the melanocortin 4 receptor (*MC4R*) is the most commonly mutated gene in human monogenic obesity [[14], [15], [16]].

MC4R is a G_s_-coupled GPCR that localizes to primary cilia [[17], [18], [19], [20]], and MC4R-expressing neurons are central regulators of energy balance and food intake [21]. In mice, genetic disruption of *Mc4r* or its known accessory protein *Mrap2,* including mutations that specifically prevent MC4R's ciliary localization, causes hyperphagic obesity [22,23]. MC4R-expressing neurons in the paraventricular nucleus of the hypothalamus (PVN^MC4R^ neurons) integrate signals from leptin-responsive neurons in the arcuate nucleus that reflect long-term energy balance [21]. PVN^MC4R^ neurons, in particular, have been shown to be necessary and sufficient for MC4R-dependent control of food intake [24,25]. Selective ablation of primary cilia from MC4R neurons or from mature PVN neurons is sufficient to impair agonist mediated MC4R activation and drive weight gain [20]. Taken together, prior work indicates that proper ciliary localization of MC4R and functioning primary cilia on MC4R neurons are essential for the weight-regulatory function of these neurons.

Curiously, some human syndromic conditions that manifest with severe obesity arise from mutations in common primary cilia genes, including the monogenic obesity gene and obesity GWAS hit *ADCY3*. Determining whether and how obesity-associated primary cilia genes function within MC4R neurons, PVN^MC4R^ neurons specifically or other defined neuronal subpopulations requires tools that enable gene perturbation *in vivo* with temporal, spatial, and cell-type specificity, as well as the ability to link gene loss with physiological outcomes.

*In vitro* systems cannot fully model specific neuronal subpopulations or capture whole-animal body weight outcomes. Standard *in vivo* approaches using conditional floxed alleles and Cre-loxP recombination lack the specificity to inactivate genes only in a mature neuronal subpopulation and lack the modularity required to interrogate multiple genes using a single mouse line [26,27]. CRISPR-based strategies offer increased specificity and flexibility; however, most implementations rely on permanent DNA modifications, which can generate truncated proteins with residual function [[28], [29], [30]], induce cytotoxic and off-target effects [[31], [32], [33], [34]], and produce mosaic editing outcomes in post-mitotic neurons [35,36]. In contrast, CRISPR interference (CRISPRi) employs an endonuclease-deficient Cas9 (dCas9) fused to a transcriptional repressor, enabling consistent, stable and non-disruptive gene silencing [34,37,38]. A Cre-dependent CRISPRi allele was recently developed by Gemberling et al. [39], but has not yet been demonstrated for post-developmental manipulation of defined neuronal subpopulations *in vivo*.

Here, we developed a Cre-dependent CRISPRi approach that enables targeted interrogation of individual obesity-associated genes in specific neuronal subpopulations *in vivo*. Applying this strategy, we refined the role of *Adcy3* in PVN^MC4R^ neurons and tested whether a subset of obesity-associated ciliopathy genes is required within MC4R neurons to maintain body-weight regulation. Specifically, we asked whether these genes are necessary for body weight regulation through MC4R neurons and whether MC4R neurons represent a site of action underlying obesity in ciliopathy patients. Together, these studies identify MC4R neurons as a key cellular locus through which multiple obesity-associated ciliary genes act and provide a framework for linking human genetic findings to defined neural circuits regulating energy balance.

## 2 Results

### 2.1 Dual component CRISPRi system enables targeted gene repression in defined neuronal subpopulations

To enable more rapid interrogation of gene function in defined neuronal populations *in vivo*, we implemented a modular Cre-dependent CRISPRi system that enables gene downregulation with simultaneous cell-type, spatial, and temporal specificity (Fig. 1). The system leverages the *Gt(ROSA)26Sor*^*tm2(CAG-dcas9/KRAB)Gers*^ allele [39] (hereafter, LSL-dCas9-KRAB), which enables Cre-dependent expression of endonuclease-deficient Cas9 fused to the KRAB transcriptional repressor (dCas9-KRAB) [39] in genetically defined neuronal populations.

**Figure 1:**
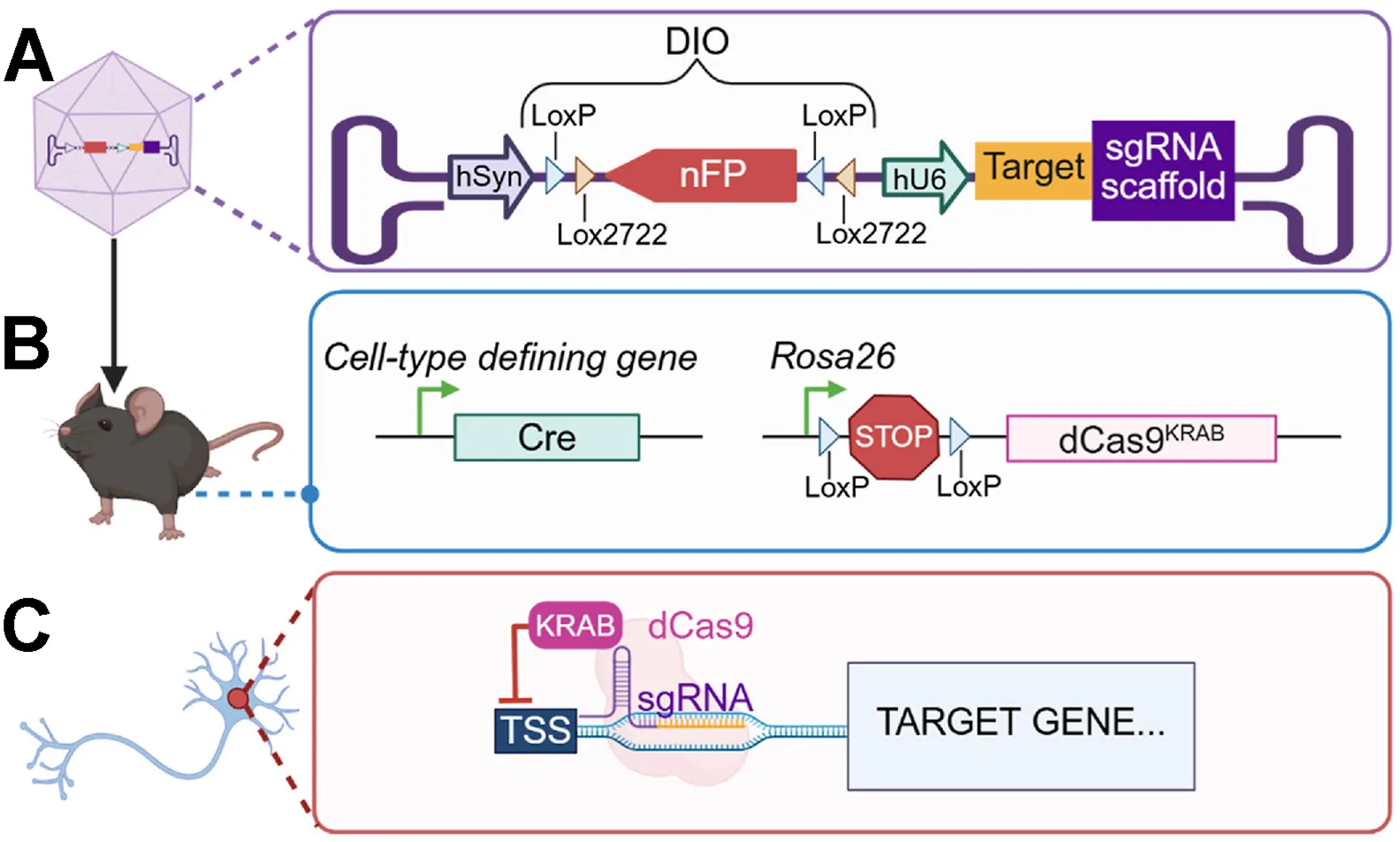
**Cre-dependent CRISPRi and AAV-delivered *sgRNA* strategy for gene repression in defined neuronal subpopulations *in vivo*. (A)** Schematic of the AAV vector encoding an sgRNA targeting the transcription start site of the downregulated gene and a Cre-dependent nuclear fluorescent reporter (DIO) driven by the human Synapsin 1 (hSyn) promoter. **(B)** Schematic of the Cre-driver allele and the Cre-dependent LSL-dCas9-KRAB allele, enabling expression of dCas9-KRAB following Cre-mediated recombination. **(C)** When Cre recombinase is expressed in a neuron, the nucleus is labelled following recombination of the delivered AAV and selective gene repression occurs due to the recombination of the LSL-dCas9-KRAB allele.

dCas9-KRAB is directed to the gene of interest following delivery of an sgRNA targeting the transcription start site via AAV injection, providing spatial and temporal control of gene repression. Stereotaxic injection enables nucleus-specific repression in adults, whereas neonatal intracerebroventricular (ICV) injection induces widespread postnatal downregulation [40]. While the sgRNA is constitutively expressed from the U6 promoter in AAV-transduced cells, CRISPRi-mediated gene repression is restricted to neurons that both express Cre recombinase, enabling dCas9-KRAB expression, and receive the AAV-delivered sgRNA. The AAV vector also encodes a Cre-dependent nuclear fluorescent reporter, enabling identification of nuclei exposed to both components (Fig. 1).

To validate the specificity of this approach, we performed immunostaining for the FLAG-tagged dCas9-KRAB protein in *Mc4r*^*t2aCre*^ LSL-dCas9-KRAB mice [20,39] following PVN-specific AAV delivery of an sgRNA and Cre-dependent KASH-GFP reporter. We observed strong nuclear co-localization of dCas9-KRAB and KASH-GFP in the PVN. In contrast, brain regions lacking Cre expression, such as the suprachiasmatic nucleus (SCN), showed no detectable signal, while regions containing MC4R neurons but lacking AAV infection, such as the nucleus of the lateral olfactory tract (nLOT), exhibited dCas9-KRAB expression without nuclear localization and no KASH-GFP signal (Fig. 2).

**Figure 2:**
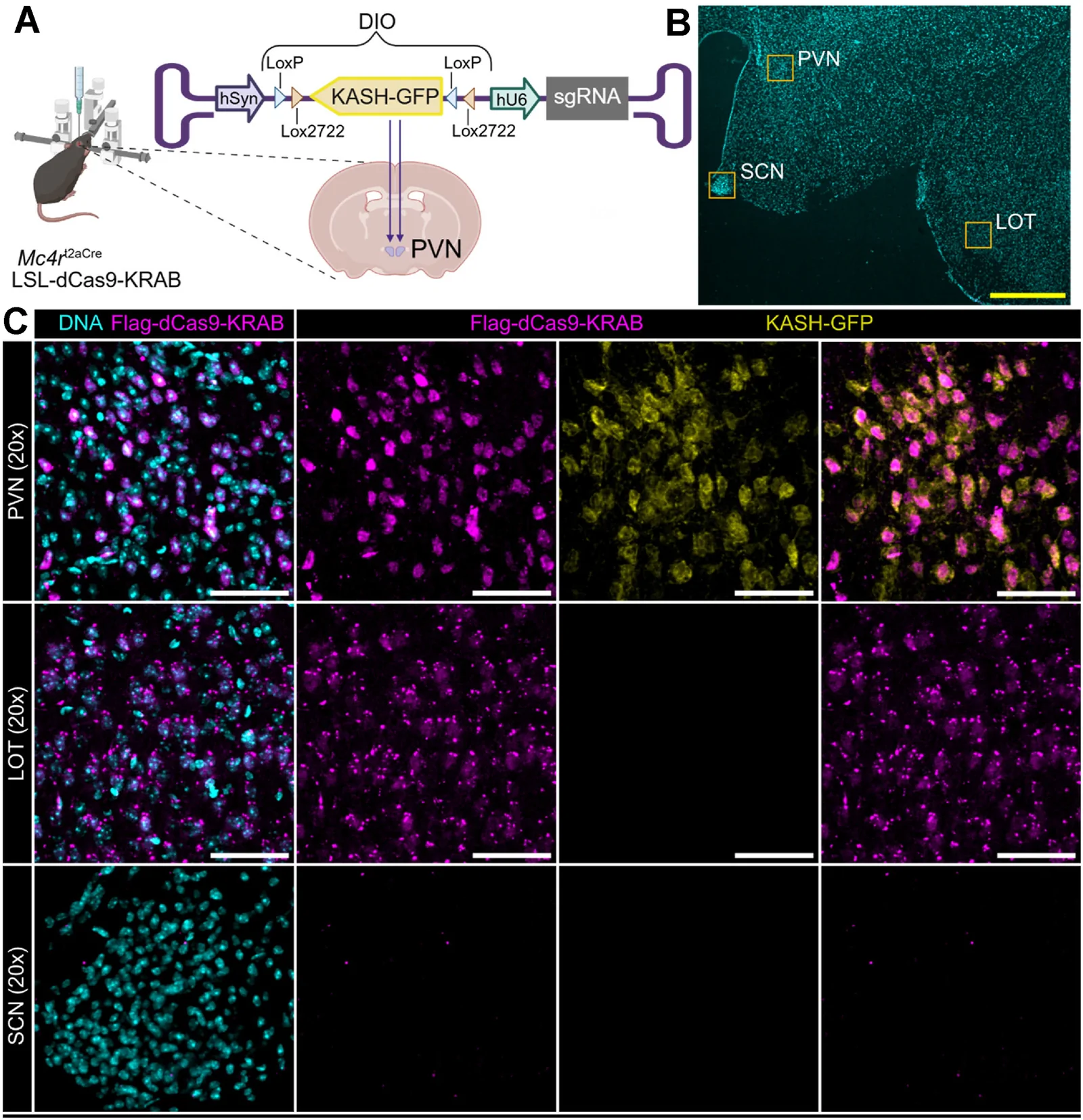
**Cre- and AAV-dependent specificity of CRISPRi targeting in MC4R neurons.** **(A)** Schematic of stereotaxic AAV injection targeting the paraventricular nucleus (PVN) of adult *Mc4r*^*t2aCre*^ LSL-dCas9-KRAB mice with a Cre-dependent KASH-GFP reporter and a non-targeting (NT) sgRNA. **(B)** Low-magnification (4 × ) coronal section showing anatomical regions, including the PVN, suprachiasmatic nucleus (SCN), and nucleus of the lateral olfactory tract (nLOT). Scale bar, 500 μm; bregma ≈ −0.58 mm. **(C)** Representative high-magnification (20 × ) images of the PVN, nLOT, and SCN showing FLAG–dCas9–KRAB and KASH-GFP signals. Nuclear co-localization is observed in the PVN, whereas regions lacking Cre expression (SCN) or AAV transduction (nLOT) show no co-localization. Scale bars, 50 μm.

### 2.2 Functional validation of Cre-dependent CRISPRi and AAV-delivered sgRNA strategy in PVN^MC4R^ neurons

To functionally validate this system, we stereotaxically injected an AAV encoding Cre-dependent nuclear mScarlet (nRFP) with either an *Mc4r*-targeting or non-targeting (NT) sgRNA into the PVN of adult *Mc4r*^*t2aCre*^ LSL-dCas9-KRAB littermates (Figure 3A). Eleven weeks post-injection, mScarlet-positive PVN nuclei were isolated by fluorescence-activated nuclei sorting (FANS) (Fig. 3C). RT-qPCR analysis of sorted nuclei revealed a significant reduction in *Mc4r* transcript in mice receiving the *Mc4r*-targeting sgRNA compared to controls (Fig. 3D), confirming effective transcriptional repression.

**Figure 3:**
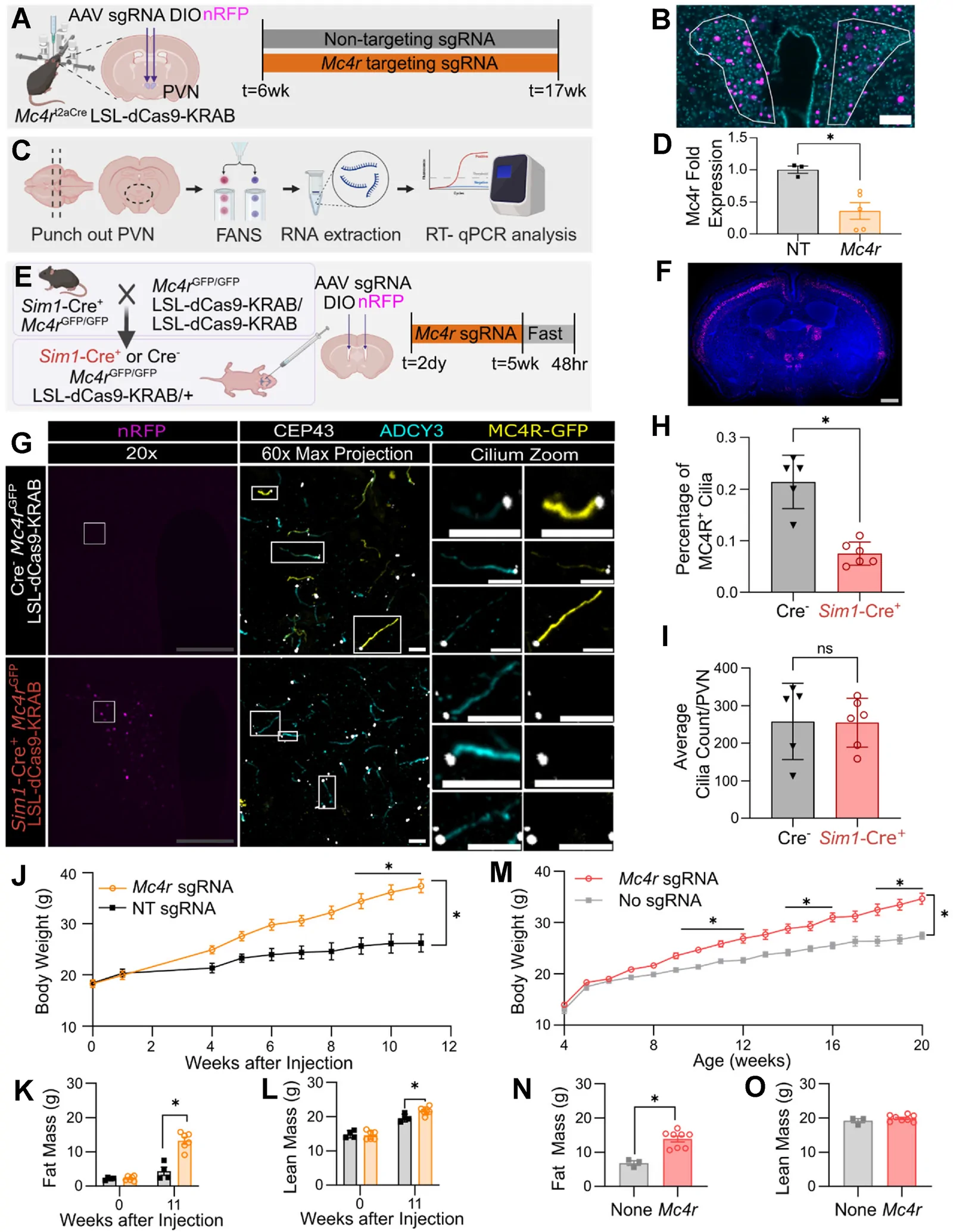
**CRISPRi-mediated *Mc4r* repression in PVN^MC4R^neurons recapitulates *Mc4r* loss-of-function obesity. (A)** Schematic of stereotaxic AAV injection into the PVN of adult *Mc4r*^*t2aCre*^ LSL-dCas9-KRAB mice, with Cre-dependent nRFP and either *Mc4r*-targeting or NT sgRNA. **(B)** Representative 20 × image of the PVN (outlined) showing nRFP expression in Cre^+^ neurons. Scale bar, 50 μm. **(C)** Workflow for recovery of nRFP^+^ nuclei by FANS, followed by RNA extraction and RT-qPCR analysis. **(D)** RT-qPCR analysis of *Mc4r* transcript levels in nRFP ^+^ nuclei (n = 6 *Mc4r* sgRNA; n = 4 NT sgRNA; unpaired t-test, ⁎p < 0.05). **(E)** Schematic of neonatal (P2) ICV injection of *Mc4r*-targeting sgRNA into *Sim1-*Cre ^+^*Mc4r*^*GFP/GFP*^ LSL-dCas9-KRAB pups or Cre^−^ littermates. **(F)** Representative 20 × coronal section containing the PVN showing nRFP expression in Cre^+^ neurons. Scale bar, 1 mm; bregma ≈ −0.82 mm. **(G)** Representative confocal images (20 × and 60 × ) showing Cre-dependent nRFP in the PVN and reduced MC4R-GFP localization to primary cilia (marked by ADCY3 and CEP43) in *Sim1-*Cre^+^ mice (n = 6 *Sim1-*Cre^+^; n = 4 Cre^−^). Scale bars, 50 μm; 5 μm. **(H)** Quantification of the proportion of MC4R-GFP ^+^ cilia per PVN in *Sim1-Cre*^*+*^ and Cre^−^ mice (n = 6 *Sim1-*Cre^+^; n = 5 Cre^−^; unpaired t-test, ⁎p < 0.05). **(I)** Total cilia number per PVN is unchanged between groups (n = 6 *Sim1*-Cre^+^; n = 5 Cre^−^; unpaired t-test, ⁎p < 0.05). **(J**–**L)** Body weight (J), fat mass (K), and lean mass (L) of female *Mc4r*^*t2aCre*^ LSL-dCas9-KRAB mice injected with *Mc4r*-targeting or NT sgRNA over 11 weeks post-injection (n = 6 *Mc4r* sgRNA; n = 4 NT sgRNA; two-way repeated-measures ANOVA with Šídák's multiple comparisons test for (J), unpaired t-test for (K–L), ⁎p < 0.05). **(M**–**O)** Body weight (M), fat mass (N), and lean mass (O) of female *Sim1*-Cre ^+^*Mc4r*^*GFP/GFP*^ LSL-dCas9-KRAB mice injected with *Mc4r*-targeting sgRNA at P2 compared to un-injected controls (n = 8 *Mc4r* sgRNA; n = 3 control; mixed-effects model (REML) for (M), unpaired t-test for (N–O), ⁎p< 0.05).

To assess the effect of *Mc4r* downregulation at the protein level, we utilized a GFP-tagged *Mc4r* allele (*Mc4r*^GFP^) to visualize MC4R localization to primary cilia [19]. Homozygous *Mc4r*^GFP^ LSL-dCas9-KRAB mice were crossed with *Sim1*-Cre mice [24] to enable dCas9-KRAB expression in PVN neurons, including PVN^MC4R^ neurons. After neonatal (P2) ICV injection of AAV encoding an *Mc4r*-targeting sgRNA and Cre-dependent nRFP (Fig. 3E), *Sim1*-Cre^+^ but not Cre^−^ littermates exhibited nRFP expression in the PVN (Fig. 3F–G). Compared to the focal adult PVN stereotaxic injections, neonatal ICV delivery produced broader brain distribution of the AAV due to both the developmental timing and ventricular route of administration. High-resolution imaging revealed an approximately 75% reduction in the proportion of MC4R-GFP ^+^ cilia per PVN of the *Sim1*-Cre^+^ mice relative to controls with no change in total cilia number per PVN (Fig. 3H–I). These results demonstrate that CRISPRi-mediated repression achieves coordinated reduction of both transcript and protein *in vivo*.

In the adult *Mc4r*^*t2aCre*^ LSL-dCas9-KRAB model with focal stereotaxic AAV delivery to the PVN, *Mc4r* downregulation resulted in significant and progressive body weight gain relative to controls (Fig. 3J). EchoMRI analysis showed that this increase was driven primarily by increased fat mass, with a smaller increase in lean mass (Fig. 3K-l), consistent with published models of PVN-specific *Mc4r* knockdown [25]. These findings indicate that CRISPRi-mediated repression of *Mc4r* in PVN^MC4R^ neurons is sufficient to recapitulate key physiological features of *Mc4r* loss-of-function obesity.

Similarly, neonatal *Mc4r* repression in *Sim1*-Cre^+^ neurons, including PVN neurons, resulted in increased body weight and fat mass relative to controls (Fig. 3M-o), providing complementary evidence that reduced MC4R expression produces physiologically relevant changes in energy balance. Because neonatal ICV delivery resulted in broad AAV distribution, contributions from Sim1-expressing MC4R populations outside the PVN cannot be excluded.

### 2.3 Systematic *in vitro* pre-screening identifies effective sgRNAs

A major advantage of this Cre-dependent CRISPRi and AAV-delivered sgRNA strategy is the ability to interrogate multiple genes within a specific neuronal subpopulation by altering only the AAV-encoded sgRNA sequence, without generating additional mouse lines. However, sgRNA choice is critical for CRISPRi efficacy, including among sgRNAs that are computationally predicted from machine learning approaches [41,42]. This variability is particularly relevant for the LSL-dCas9-KRAB allele, which features a KRAB domain derived from KOX1, reported to be sensitive to sgRNA selection than other KRAB domains [41,43].

To ensure robust *in vivo* gene repression, we performed systematic in *vitro* pre-screening of candidate sgRNAs using Neuro2a (N2a) cells stably expressing the same dCas9-KRAB construct as the LSL-dCas9-KRAB allele. Across 20 candidate genes, we tested 80 sgRNAs (2–5 per gene) and identified 26 sgRNAs that produced effective knockdown across 16 genes (Supplementary Table 1). SgRNAs were selected from established CRISPRi libraries [42] or designed using CRISPick [44,45], and the most effective sgRNA for each gene achieved knockdown efficiencies ranging from 79% to 84%. For four genes, no effective sgRNA was identified.

This pre-screening step was essential to ensure consistent and efficient gene repression *in vivo* and to enable reliable comparison of phenotypic outcomes across targeted genes.

### 2.4 ADCY3 is required in adult PVN^MC4R^ neurons for normal body-weight regulation

ADCY3 is a ciliary adenylyl cyclase that generates cAMP downstream of GPCR signaling and is widely used as a marker of neuronal primary cilia [[46], [47], [48]]. Given that MC4R localizes to the primary cilium [19], ADCY3 has been proposed to function as a downstream effector of MC4R signaling [20,46,[49], [50], [51]]. Consistent with this, *ADCY3* loss-of-function mutations in humans and mice result in obesity [[52], [53], [54], [55], [56], [57]]. However, multiple adenylyl cyclases have been reported to localize to neuronal cilia [58] or to have high hypothalamic expression [59], leaving it unclear whether ADCY3 specifically is required for body-weight regulation in MC4R neurons. In addition, because ADCY3 is broadly expressed throughout the adult mouse brain [47], it is unknown whether its effects on body weight are mediated through MC4R neurons or other neuronal populations.

Using our Cre-dependent CRISPRi and AAV-delivered sgRNA strategy, we therefore addressed two key questions: (1) whether ADCY3 is specifically required for the weight regulatory function of adult PVN^MC4R^ neurons and (2) whether selective downregulation of *Adcy3* in PVN^MC4R^ neurons is sufficient to perturb body-weight regulation.

We stereotaxically injected AAV encoding Cre-dependent nRFP and either a pre-screened *Adcy3*-targeting or non-targeting (NT) sgRNA into the PVN of adult *Mc4r*^*t2aCre*^ LSL-dCas9-KRAB littermates (Fig. 4B). A subset of mice from both groups were co-injected with a Cre-dependent, cilia-localized fluorescent reporter to identify primary cilia in CRISPRi-treated MC4R neurons for analysis of ciliary ADCY3 (Fig. 4A). High-resolution confocal imaging 12 weeks post-injection revealed a substantial reduction in ADCY3 immunofluorescence specifically in MC4R^+^ cilia in *Adcy3*-sgRNA–treated mice (Fig. 4C–D), confirming effective protein-level repression in the targeted neuronal subpopulation.

**Figure 4:**
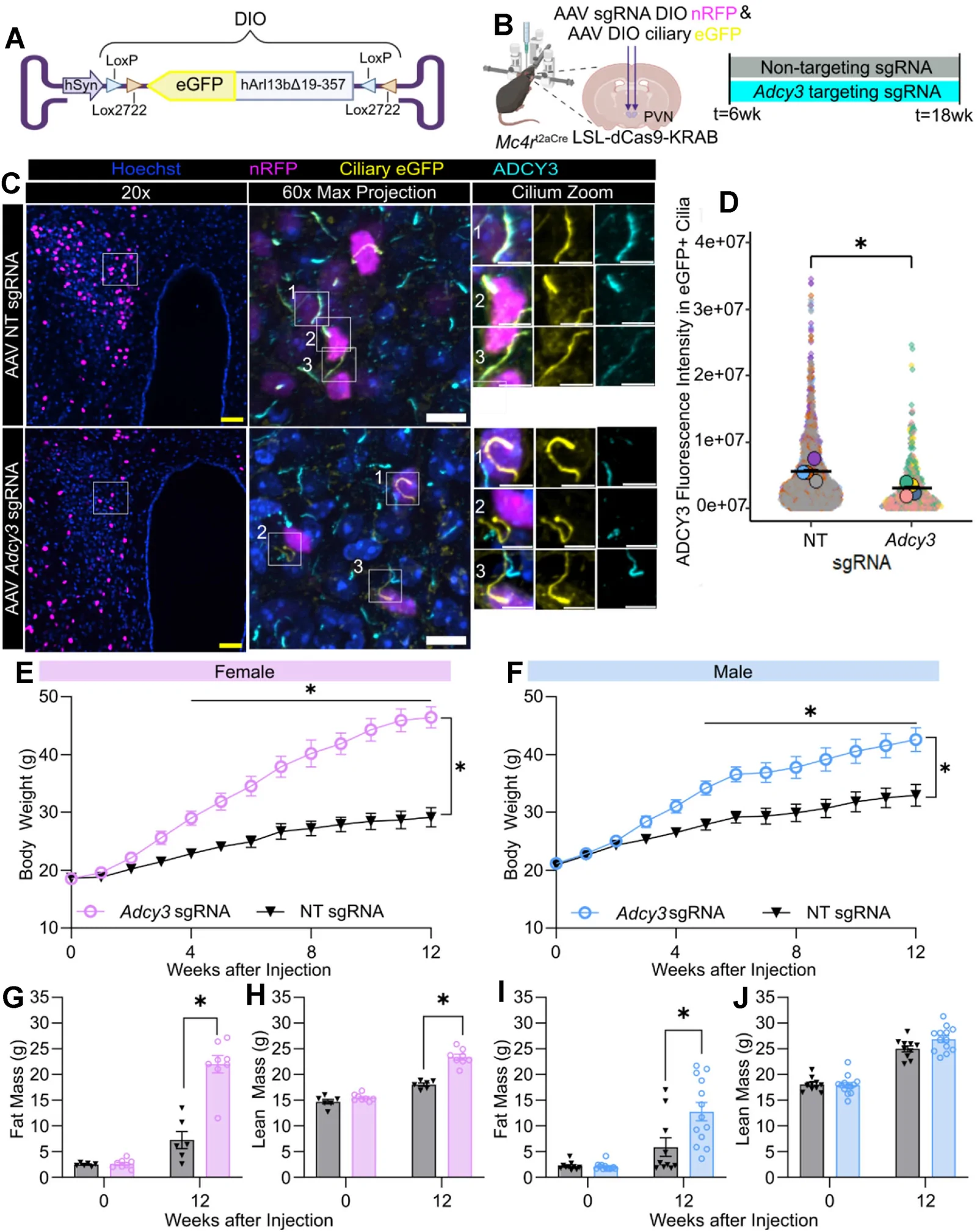
**CRISPRi-mediated *Adcy3* repression in adult PVN^MC4R^neurons increases body weight and adiposity. (A)** Schematic of the Cre-dependent AAV reporter used to label primary cilia in MC4R neurons. The reporter consists of GFP fused to a ciliary targeting sequence derived from human ARL13B, expressed under the hSyn promoter in DIO orientation, enabling Cre-dependent fluorescent labeling of primary cilia **(B)** Experimental design: adult *Mc4r*^*t2aCre*^ LSL-dCas9-KRAB mice received bilateral PVN injections of AAV encoding Cre-dependent nRFP and either *Adcy3*-targeting or NT sgRNA. A subset of mice was co-injected with a Cre-dependent cilia-localized eGFP reporter. **(C)** Representative confocal images of the PVN showing MC4R^+^ nuclei (nRFP), primary cilia (eGFP), and ADCY3 immunostaining. Insets show reduced ADCY3 signal in MC4R^+^ cilia following *Adcy3* sgRNA treatment compared to NT controls. Scale bars, 50 μm; 5 μm **(D)** Quantification of ADCY3 fluorescence intensity in MC4R^+^ cilia (n = 4 mice per group). Each point represents a single cilium; larger points indicate mean values per animal; bars indicate group means. **(E**–**F)** Body weight trajectories of female (E) and male (F) mice over 12 weeks post-injection (females: n = 10 *Adcy3* sgRNA, n = 9 NT; males: n = 16 *Adcy3* sgRNA, n = 12 NT; two-way repeated-measures ANOVA with Šídák's multiple comparisons test, ⁎p < 0.05). **(G**–**J)** Body composition assessed by EchoMRI at baseline and 12 weeks post-injection. *Adcy3* repression increased fat mass in females (G) and males (I) and increased lean mass in females (H) (females: n = 8 *Adcy3* sgRNA, n = 6 NT; males: n = 13 *Adcy3* sgRNA, n = 10 NT; unpaired t-test, ⁎p < 0.05).

Physiologically, *Adcy3* repression resulted in significant and progressive weight gain over 12 weeks (Fig. 4E–F). EchoMRI analysis showed that this increase was primarily attributable to increased fat mass in both sexes, with a smaller increase in lean mass (Fig. 4G–J).

These findings resolve both questions: ADCY3 is specifically required for normal body-weight regulation in PVN^MC4R^ neurons, and its selective downregulation in this neuronal population alone is sufficient to drive obesity.

### 2.5 MC4R neurons are a convergent site of action for obesity-associated ciliopathy genes

Primary cilia are present on most mammalian cells [60], and mutations in genes required for ciliary structure or function cause pleiotropic syndromic conditions known as ciliopathies [61]. Several ciliopathies manifest with obesity, including Bardet-Biedl Syndrome (BBS), which results from mutations in the 20+ proteins that compose and regulate the ciliary trafficking complex known as the BBSome [[62], [63], [64]], and Impaired Intellectual Development, and Truncal Obesity, Retinal Dystrophy, and Micropenis Syndrome (MORMS) arising from certain mutations in *INPP5E* that prevent its ciliary localization [[65], [66], [67]]. These observations implicate primary cilia in energy homeostasis. Given that proper ciliary localization of MC4R is essential for its role in body-weight regulation, we hypothesized that obesity-associated ciliary genes act, at least in part, through MC4R neurons.

To test this, we performed an *in vivo* CRISPRi screen targeting a subset of obesity-associated ciliary genes in MC4R-expressing neurons (Fig. 5A). We focused on genes with prior evidence linking them to body-weight regulation, including *Ankrd26*, *Bbs10*, and *Inpp5e.* ANKRD26 is a transition fiber protein thought to gate GPCR entry into the primary cilium [68]; *Ankrd26* ^−/−^ mice are heavier than wild-type littermates and show reduced ADCY3 accumulation in PVN cilia [69,70]. *Bbs10* encodes a chaperone protein that assembles the BBSome complex regulating exit of receptors and signaling proteins from the primary cilia [71,72], and together with *Bbs1*, accounts for roughly 40% of Bardet–Biedl syndrome (BBS) cases [63,71]. *Bbs1*^−/−^ mice are obese [73], while *Bbs10* is comparatively understudied. INPP5E, a ciliary transition zone inositol polyphosphate-5-phosphatase, is important for cilia compartmentalization and signaling [74]; its tamoxifen-induced knockout in 4-week-old mice also leads to obesity, consistent with MORM syndrome [65].

**Figure 5:**
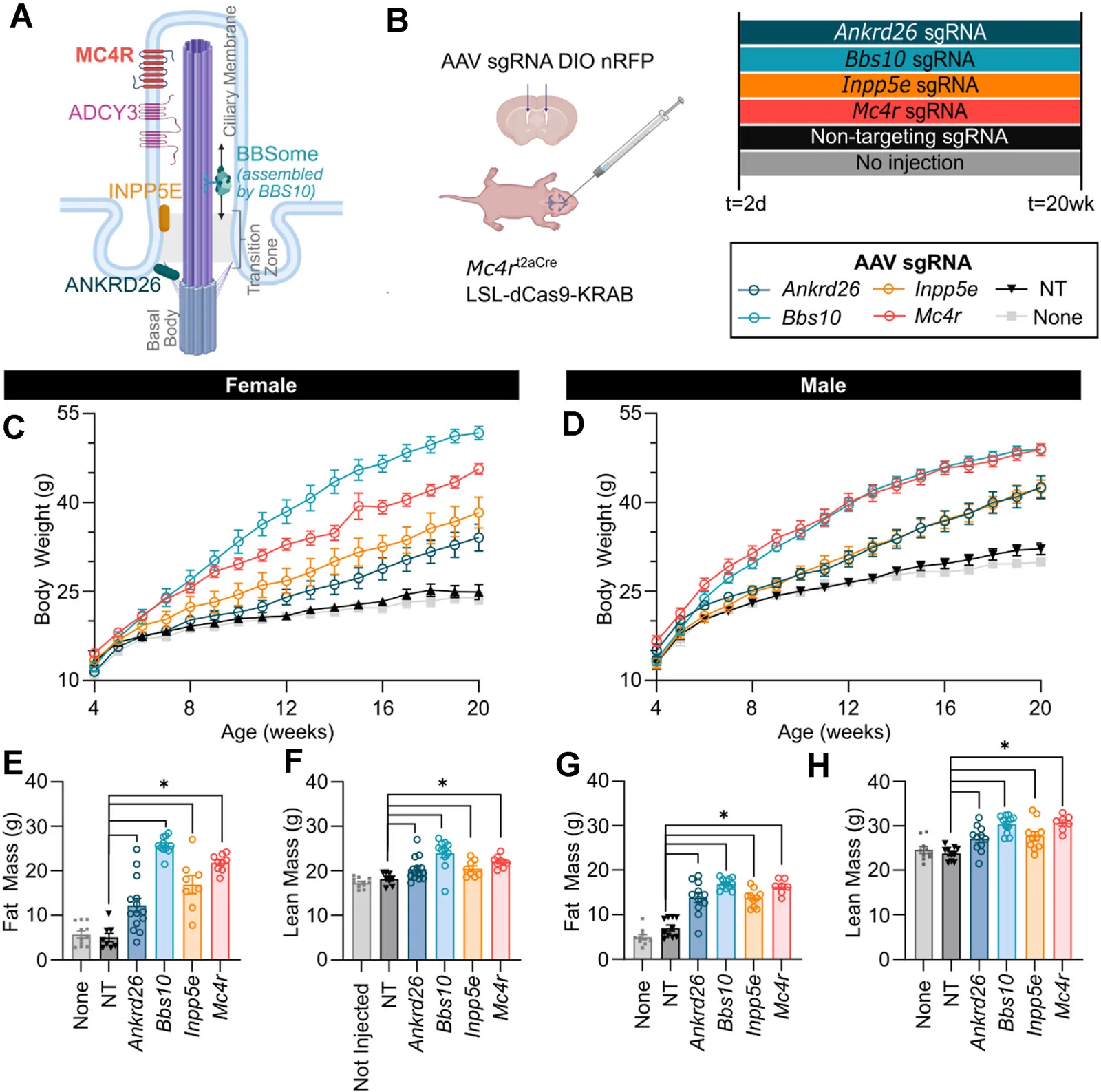
***In vivo* CRISPRi screen identifies obesity-associated ciliopathy genes required in MC4R neurons. (A)** Schematic of obesity-associated ciliopathy proteins localized to distinct sub compartments of the primary cilium in MC4R neurons. **(B)** Schematic of neonatal *in vivo* CRISPRi screen. *Mc4r*^*t2aCre*^LSL-dCas9-KRAB neonates (P0–P2) received ICV injections of AAV encoding Cre-dependent nRFP and sgRNAs targeting *Ankrd26, Bbs10*, or *Inpp5e*. Control groups included *Mc4r*-targeting (positive control), non-targeting (NT), and uninjected mice. **(C**–**D)** Body weight trajectories of female (C) and male (D) mice from 4 to 20 weeks of age following gene repression (females: n = 14 *Ankrd26*, 11 *Bbs10*, 8 *Inpp5e*, 9 *Mc4r*, 8 NT, 11 uninjected; males: n = 11 *Ankrd26*, 11 *Bbs10*, 10 *Inpp5e*, 7 *Mc4r*, 11 NT, 10 uninjected; mixed-effects model (REML) with Šídák's multiple comparisons test; p values in Supplementary Table 2–3). **(E**–**H)** Fat and lean mass in females (E, F) and males (G, H) at 20 weeks measured by EchoMRI (same sample sizes as in (C–D); unpaired t-test, ⁎p < 0.05).

Neonatal *Mc4r*^*t2aCre*^ LSL-dCas9-KRAB pups received ICV injections of AAV encoding Cre-dependent nRFP and sgRNAs targeting individual genes (Fig. 5B). Negative controls received a non-targeting sgRNA or no injection, whereas positive controls received the *Mc4r*-targeting sgRNA. Non-targeting and uninjected controls did not differ in body weight or composition, while *Mc4r*-targeted controls exhibited the expected obesity phenotype, with increased body weight and both fat and lean mass (Fig. 5C–H). Downregulation of *Bbs10* produced weight gain comparable to that observed with *Mc4r* repression in both sexes, with corresponding increases in fat and lean mass. Downregulation of *Ankrd26* or *Inpp5e* also resulted in significant weight gain, although to a lesser extent (Fig. 5C–H).

Overall, these results demonstrate that multiple obesity-associated ciliopathy genes are required for normal body-weight regulation in MC4R neurons and identify MC4R neurons as a convergent site through which these genes act to control energy balance.

## 3 Discussion

Understanding how obesity-associated genes act within defined neuronal populations remains a major barrier to linking human genetics to mechanisms of energy balance. Here, we identify MC4R neurons as a convergent locus through which multiple obesity-associated ciliary genes regulate body weight. We show that *Adcy3* is specifically required for the weight-regulatory function of adult PVN^MC4R^ neurons and that its selective downregulation in this population is sufficient to drive obesity. In addition, we demonstrate that multiple ciliopathy-associated genes, including *Ankrd26, Bbs10*, and *Inpp5e*, act within MC4R neurons to control energy balance, indicating that dysfunction in this neuronal population contributes to obesity associated with impaired primary cilia function. These findings establish that *Adcy3* is necessary for normal PVN^MC4R^ neuronal function and that related ciliary genes are required for normal MC4R neuronal function. Disruption of these genes within this population is sufficient to perturb body-weight regulation. However, definitive demonstration of sufficiency will require complementary gain-of-function or rescue experiments, such as re-expression of these genes in adult PVN^MC4R^ neurons.

The body-weight phenotypes observed here are consistent with established MC4R loss-of-function models, and comparison across these studies highlights how anatomical and mechanistic restriction shape the onset and severity of obesity. Congenital whole-body *Mc4r* loss produces the earliest and most severe obesity phenotype [22], but broad neonatal *Mc4r* repression in MC4R-expressing neurons ultimately approached a similar magnitude of weight gain with a more gradual development. This difference may reflect both extent of gene disruption and timing, including contributions from broader MC4R populations and developmental effects. Adult PVN-specific *Mc4r* repression closely recapitulated the phenotype of previous PVN-specific *Mc4r* deletion [25]. Disruption of ciliary signaling within adult PVN^MC4R^ neurons produced a body-weight phenotype similar in magnitude and time course to direct PVN-specific *Mc4r* loss. Previous inhibition of ciliary adenylyl cyclase activity in PVN^MC4R^ neurons resulted in approximately 6 g of excess weight gain by 5 weeks after manipulation [20], while *Adcy3* repression in our study produced a 5–6 g difference from controls at the same time point. In general, neonatal repression of additional cilia-associated genes in MC4R neurons produced progressive obesity of varying severity, in some cases approaching the magnitude of congenital whole-body Mc4r loss. This variability may reflect the distinct roles of these proteins in ciliary structure, trafficking, and signaling, as well as differences in the extent of gene repression.

Although dysfunction of MC4R (or PVN^MC4R^) neurons contributes to the obesity phenotype observed in global gene loss, these neurons are unlikely to be the only site of action. In the case of *Adcy3*, studies in other hypothalamic regions suggest that multiple neuronal populations contribute to the metabolic effects of ADCY3 deficiency. In the ventromedial nucleus of the hypothalamus (VMH), disruption of *Adcy3* either causes obesity [75] or increases susceptibility to high-fat diet [76], while expression of a constitutively active ADCY3 construct in MC4R-expressing neurons of the dorsomedial hypothalamic nucleus (DMH^MC4R^ neurons) promotes leanness [77]. With our PVN^MC4R^ findings, these studies suggest that distinct neuronal subpopulations can each contribute to energy homeostasis, and their combined dysfunction likely underlies the phenotypes observed in whole-body *Adcy3* deficiency. Similarly, other neuronal populations are likely to contribute to ciliopathy-associated obesity.

Mechanistically, we have previously demonstrated that loss of primary cilia or inhibition of ciliary adenylyl cyclase signaling in PVN^MC4R^ neurons impairs agonist mediated MC4R activity. However, *Adcy3, Ankrd26, Bbs10,* and *Inpp5e* may influence MC4R neuron function through several pathways, including altered trafficking or retention of MC4R or ADCY3 in the primary cilium, disruption of ciliary second messenger signaling, or compromised ciliary structural integrity. These mechanisms are not mutually exclusive and may differentially affect signaling dynamics within the cilia of MC4R neurons. ADCY3 likely represents a key downstream effector within this pathway, while other ciliopathy-associated genes may act upstream by regulating ciliary localization or signaling. Combining gene repression with cilia reporters or GFP-tagged MC4R provides a means to assess perturbation effects on ciliary structure and receptor localization, and co-downregulation experiments may reveal epistatic interactions or functional redundancy among cilia-associated genes. Such studies could also help define how individual ciliary components contribute to MC4R signaling, distinguish defects that preserve versus impair responsiveness to receptor agonism, and ultimately inform which genetic forms of cilia-associated obesity may be amenable to MC4R-targeted therapies.

More broadly, these mechanisms are likely to extend beyond MC4R neurons. MC4R is not the only ciliary GPCR involved in CNS energy balance [[78], [79], [80], [81]], and neurons expressing other weight-relevant ciliary GPCRs are plausibly additional sites of action for ciliopathy-associated obesity. Primary cilia also play essential roles in neuronal subtype specification, migration, axon targeting, and synapse formation, raising the possibility that gene loss at different developmental stages may produce distinct circuit and behavioral outcomes [82,83]. The temporal and regional flexibility of Cre-dependent CRISPRi and AAV-delivered sgRNA enables separation of developmental and adult homeostatic functions of primary cilia genes.

This strategy also enables both perturbation and rescue. Pairing CRISPRi with CRISPRa or re-expression strategies will allow testing of sufficiency and functional assessment of specific human variants. Because CRISPRi permits repression of non-coding regulatory elements, it provides a framework for interrogating intronic or intergenic GWAS loci in defined neuronal populations.

As with any CRISPR-based strategy, performance depends on sgRNA design [43] and AAV targeting. SgRNA efficacy varies across genes and should be considered when comparing phenotypic outcomes. In addition, CRISPRi-mediated repression may not fully recapitulate complete loss-of-function, and residual gene expression could influence the magnitude of observed phenotypes. Although pre-screening in N2a cells improves *in vivo* performance, this approach is limited to genes expressed in that cell line, underscoring the need for broader resources of empirically validated mouse CRISPRi guides. Spatial specificity of the CRISPRi system also depends on AAV targeting, and viral spread beyond the intended region may affect nearby Cre-expressing populations.

In summary, our work establishes MC4R neurons as a convergent neuronal locus for multiple obesity-associated ciliary genes and clarifies *Adcy3*'s cell-type–specific role in adult PVN^MC4R^ neurons. These findings provide a mechanistic link between primary cilia signaling and central regulation of energy balance, offer a framework for linking human genetic variation to defined neuronal populations, and present a potential cellular explanation for obesity observed in patients with ciliopathies. Extending this approach across additional genes and neuronal populations will further elucidate the circuits and mechanisms underlying obesity and facilitate translation of human genetic discoveries into cellular and circuit-level insight.

## 4 Materials and methods

### 4.1 Mice and physiological measurements

All mice were housed in a barrier facility under a 12 h light/dark cycle (lights on at 07:00) at 23 ± 2 °C with 50–70% relative humidity. Animals were group-housed (up to five per cage) and provided with ad libitum access to rodent diet 5058 (LabDiet) and water. All procedures were approved by the UCSF Institutional Animal Care and Use Committee and conducted in accordance with National Institutes of Health guidelines.

Mouse Lines.

• *Mc4r*^*tm2(t2a Cre)Vai*^ (*Mc4r*^*Cre*^): t2a ribosomal skip sequence enabling co-expression of Cre-recombinase and endogenous MC4R [20].
• *Gt(ROSA)26Sor*^*tm2(CAG-cas9*^*⁎*^*/ZNF10*^*⁎*^*)Gers*^ (LSL-dCas9-KRAB): Cre-dependent expression of dCas9-KRAB following removal of lox-stop-lox cassette (JAX #033066) [39].
• *Mc4r*^*tm1(egfp)Vai*^ (*Mc4r*^*GFP*^): endogenous MC4R expressed with C-terminal EGFP tag [19].
• *Tg(Sim1-cre)1Lowl* (*Sim1*-Cre): Cre expression under control of the *Sim1* promoter (JAX #006451) [24].

All lines were maintained on C57BL/6 J background. Genotyping was performed using PCR- or qPCR-based assays according to standard protocols (primer and probe sequences available upon request). Both male and female mice were used for physiological experiments. Animals were weaned at 4 weeks of age, group-housed and weighed weekly; body composition was assessed every 4–6 weeks using EchoMRI.

### 4.2 Plasmids

The sgRNA AAV vector was derived from AAV-phsyn-FLEX-KASH-EGFP-U6-shRNA (Addgene plasmid # 129,708, a gift from Yi Zhang) [84]. The shRNA scaffold was replaced by the sgRNA expression cassette (minimal humanU6 promoter with *Streptococcus pyogenes* sgRNA scaffold) from the U6-sgRNA-CMV-mCherry-WPREpA plasmid, a gift from N. Ahituv previously described in Matharu et al. [85]. The backbone was digested with SapI and PmlI, and the sgRNA cassette was inserted using NEB HiFi DNA Assembly. KASH-EGFP was replaced by 3xnls-mScarlet from 3xnls-mScarlet-I (Addgene plasmid # 98,816, a gift from Dorus Gadella) [86] using NEB HiFi DNA Assembly after digestion with AscI and NheI. sgRNA AAV vectors targeting individual genes were generated following NEB's protocol for bridging double-stranded DNA with a single-stranded DNA oligo using NEBuilder HiFi DNA Assembly after SapI restriction digest.

The lentiviral vector used for sgRNA pre-screening was pLG1 library vector (pU6-sgRNA Ef1α-Puro-T2A-mCherry) (Addgene plasmid #217306), originally deposited by Luke Gilbert. Individual target sequences were inserted by ligating annealed oligonucleotides into the vector after digestion with BstXI and BlpI.

### 4.3 sgRNA pre-screening in N2a-dCas9-KRAB cells

Neuro-2a cells (ATCC, CCL-131) were dissociated using 0.25% trypsin, counted, and seeded at a density of 3 × 10^5^ cells per well. Cells were transduced with lentivirus encoding CRISPRi (dCas9-BFP-KRAB (Kox1); plasmid pMH0001, Addgene #85969) in complete medium supplemented with polybrene (final concentration ∼6–8 μg/mL), with virus added directly to the cells. Cells were incubated at 37 °C, and transduction efficiency was assessed 48 h post-infection by flow cytometry based on BFP expression. Following transduction, cells were allowed to recover and expand for two weeks, after which they were bulk sorted and enriched by fluorescence-activated cell sorting (FACS) using a Sony SH800S Cell Sorter. Sorted populations were expanded for downstream experiments. Flow cytometry data were analyzed using FlowJo™ (v10.10).

N2a cells stably expressing dCas9-KRAB were cultured in DMEM [DMEM (Gibco, 11,995,065) supplemented with 10% FBS (Gibco, A5670801) and 1% penicillin–streptomycin (Sigma–Aldrich, P4333)]. Lentivirus was made by the UCSF ViraCore (titer 1.0–4.5 × 10^5^ vg/mL). For reverse transduction, 65 μL of lentivirus prep and 35 μL of DMEM was added to tissue-culture treated 96-well plate resulting an MOI range of 0.3–1.5 with a mean MOI ∼0.75.20,000 cells in 100 μL of DMEM with 16 μg/mL Polybrene (EMD Millipore Core, TR-1003-G) were added directly to virus-containing wells. Plates were incubated at 37 °C, 5% CO_2_ for 48 h.

Puromycin (Gibco, A11138-03) was added 48 h post-transduction to a final concentration of 2 μg/mL, and cells were selected for 72 h; puromycin-containing medium was refreshed after 48 h. Following selection, cells were allowed to recover for an additional 72 h before RNA collection. Infection efficiency was assessed by mCherry fluorescence using flow cytometry (Invitrogen Attune NxT Flow Cytometer), with debris and doublets excluded; gating was based on unstained and single-color controls. Across samples, the mean infection efficiency following puromycin selection was ∼85%.

RNA was extracted for downstream analysis as described in RNA Extraction, cDNA Synthesis, and Quantitative PCR. Knockdown efficiency for each sgRNA was calculated relative to pooled controls. Positive control sgRNAs targeting *Cd81* produced ∼70% knockdown under these conditions; therefore, sgRNAs achieving ≥70% knockdown were considered successful. All sgRNAs used *in vivo* in this study achieved approximately ≥80% knockdown**.**

### 4.4 RNA extraction, cDNA synthesis, and quantitative PCR

RNA was isolated using the Qiagen RNeasy Micro Plus Kit following the manufacturer's instructions. For N2a cell homogenates, 1 μg of RNA was used for cDNA synthesis with the Qiagen QuantiTect Reverse Transcription Kit. For FANS-isolated nuclei, total RNA was below the limit of detection by NanoDrop; therefore, 6 μL of the RNA extract was used directly for cDNA synthesis. qPCR was performed using Bio-Rad iQ SYBR Green Supermix on a Bio-Rad CFX Connect Real-Time PCR Detection System. All reactions were run in technical duplicate. qPCR primers are in Supplemental Table 4.

Gene-expression values were quantified using the 2^−ΔΔCt^ method. For each sample, ΔCt was calculated as the difference between the Ct of the gene of interest and the Ct of the appropriate housekeeping gene (N2a: *Hprt*; FANS: *Gapdh*).

N2a knockdown efficiency: ΔΔCt was calculated by subtracting the ΔCt of the sgRNA well from the ΔCt of the pooled control (constructed from the two control pools not containing that sample). Percent knockdown was computed as (1 − 2^−ΔΔCt^) × 100. FANS samples *Mc4r* expression: ΔΔCt was calculated as the difference between ΔCt of mScarlet-positive nuclei from mice injected with *Mc4r*-sgRNA and ΔCt of mScarlet-positive nuclei from control-sgRNA mice.

### 4.5 AAV production, purification, and titration

#### 4.5.1 AAV DJ preparation

AAV DJ used in adult stereotaxic injections was produced in AAVpro cells (Takara, 632,273, passage number less than 10) maintained in complete DMEM [DMEM (Gibco, 11,995,065) supplemented with 10% FBS (Gibco, A5670801) and 1% penicillin–streptomycin (Sigma–Aldrich, P4333)]. Cells were seeded at 2.5 × 10^7^ cells per 15-cm dish (two dishes per virus) and transfected 24 h later with 20 μg each of pHelper (Takara), pRC DJ [87], and AAV sgRNA plasmids using 120 μl TransIT-293 reagent (Mirus, 2700) in 4 mL Opti-MEM (Gibco, 31,985–070). After 24 h, the medium was replaced with DMEM containing 2% FBS without antibiotics, and cultures were incubated for an additional 48 h before harvest. Viral extraction and purification were performed using the AAVpro Purification Kit Midi (Takara, 6675) following the manufacturer's instructions. Briefly, cells were detached with 0.5 M EDTA, pelleted, and resuspended in AAV Extraction Solution A (1 mL per dish) before lysis by vortexing and incubation at room temperature. Lysates were clarified at 4,000×*g*, mixed with AAV Extraction Solution B, treated with Cryonase, and incubated sequentially with Precipitator A and B prior to centrifugation at 9,000×*g*. Supernatants were filtered (0.45 μm) and concentrated using Amicon Ultra-4 100-kDa centrifugal filters (Millipore, UFC810008) with five buffer-exchange steps in Suspension Buffer. Viral titers were quantified using the AAVpro Titration Kit (Takara, 6233) according to the manufacturer's protocol, with samples run in duplicate.

#### 4.5.2 High-Titer AAV PHP.eB preparation

AAV PHP. eB (used in neonatal injections) was produced following Negrini et al. [88] with modifications implemented by Ramani et al. [89]. For each virus, three 15-cm dishes were seeded with 1.5 × 10^7^ AAVpro cells (Takara, 632,273, passage number less than 10) in 40 mL complete DMEM [DMEM (Gibco, 11,995,065) supplemented with 10% FBS (Gibco, A5670801) and 1% penicillin–streptomycin (Sigma–Aldrich, P4333)]. The following day, cells were transfected with a DNA–PEI mixture containing 20 μg pAdDeltaF6 (Addgene #112867; gift from James M. Wilson), 11 μg pUCmini-iCAP-PHP.eB (Addgene #103005; gift from Viviana Gradinaru [90]), 7 μg of the AAV sgRNA plasmid, and 75 μL of 1 μg/μL linear PEI (Polysciences, 23,966) in 4 mL Opti-MEM (Gibco, 31,985,062). The solution was incubated at room temperature for 10 min and then added dropwise to the cells. After 24 h, the medium was replaced with 27 mL fresh Opti-MEM mixed with 3 mL of the removed medium. At 72 h post-transfection, both cells and medium were triturated, transferred to 50-mL conical tubes, and adjusted to 30 mL with Opti-MEM.

Chloroform (3 mL) was added to each tube, vortexed for 30 s, and centrifuged at 3,000×*g* for 5 min at room temperature. The aqueous phase was transferred to a new tube, and cold 5 × AAV precipitation solution (40% PEG 8000 and 2.5 M NaCl) was added to a 1 × final concentration. Tubes were vortexed for 10 s, incubated on ice for ≥1 h, and centrifuged at 3,000×*g* for 30 min at 4 °C. Supernatants were removed, and pellets were air-dried for 10 min before resuspension in 1 mL 50 mM HEPES, 3 mM MgCl_2_. Resuspended material was treated with 1 μL DNase I (NEB, M0303S) and 10 μL RNase A (Thermo Scientific, EN0531) at 37 °C for 15 min, followed by extraction with an equal volume of chloroform (1 mL). After vortexing for 15 s and centrifugation at 16,000×*g* for 5 min, the aqueous phase was transferred to 0.5-mL Amicon Ultra filters (100-kDa cutoff; Millipore, UFC510024) in 400 μL increments and centrifuged at 14,000×*g* for 3 min. Virus obtained from the three plates corresponding to each sgRNA plasmid was pooled onto a single filter unit.

Virus preparations were buffer exchanged into 1 × DPBS by two washes using 400 μL DPBS and centrifugation (14,000×*g*, 3 min, room temperature). Final preparations were mixed thoroughly and aliquoted into 4.5 μL volumes for storage at −80 °C. Viral genome titers were determined from thawed aliquots using the AAVpro Titration Kit for Real-Time PCR (Takara, 6233) following the manufacturer's instructions.

### 4.6 AAV injection

#### 4.6.1 Adult stereotaxic injection

Six-week-old *Mc4r*^Cre^ LSL-dCas9-KRAB mice were injected bilaterally with AAV DJ produced in-house. Mice were anesthetized with 4% isoflurane (Dechra) for induction and maintained at 1–2% isoflurane during surgery, with body temperature maintained at 30–37 °C using a heating pad. Surgeries were performed under aseptic conditions using standard stereotaxic techniques. Briefly, the skull was exposed, leveled, and holes drilled at PVN coordinates (AP = −0.8, ML = ± 0.25, DV = −5.3). AAV was delivered at a rate of 0.1 μL/min for a total volume of 400 nL of 4 × 10^12^ vg/mL (Fig. 2) or 300 nL of 2.75 × 10^12^ vg/mL per site (Fig. 4). For both experiments, the non-targeting control AAV was diluted to match titer and volume. Mice received preoperative analgesia (buprenorphine, 0.3 mg/kg, Covetrus), local anesthetic (Lidocaine, 2.5 mg/kg, VetOne) and postoperative anti-inflammatory and antibiotic treatment (meloxicam, 5 mg/kg, Pivetal; enrofloxacin, 20 mg/kg, Baytril). Animals were weighed and assessed by EchoMRI prior to surgery and assigned to experimental groups to ensure weight-matched littermates. Post-injection, mice were weighed weekly, and body composition was periodically assessed. Following completion of experiments, injection accuracy was confirmed postmortem by evaluation of Cre-dependent nuclear mScarlet expression in the PVN. Mice lacking detectable PVN mScarlet expression were excluded from analysis.

#### 4.6.2 Neonatal intracerebroventricular (ICV) injections

*Mc4r*^Cre^ LSL-dCas9-KRAB P1–P2 pups were cryoanesthetized on ice until immobile. The scalp was cleaned with povidone–iodine and ethanol swab. AAV-sgRNA was diluted in 1 × DPBS with 0.1% trypan blue to a concentration of 1–2 × 10^13^ vg/mL and loaded into a 10 μL Hamilton syringe (7629–01) with a 32-gauge beveled needle (7803–04). A total of 3 μL per pup (1.5 μL per hemisphere) was injected. The needle was inserted at a point ∼2/5 of the distance from lambda to the eye and advanced 3 mm to reach the lateral ventricle. After injection, pups were placed on a warming pad with nesting material and returned to the parent cage once movement resumed.

### 4.7 Isolation of PVN^MC4R^ nuclei by fluorescence-activated nuclei sorting (FANS)

Mice (*Mc4r*^Cre^ LSL-dCas9-KRAB) injected with Cre-dependent nuclear mScarlet AAV-sgRNA were euthanized, and brains were rapidly removed and rinsed in ice-cold PBS. Brains were placed ventral side up in a chilled brain matrix, and a coronal slice containing the hypothalamus was collected. A tissue punch was taken to isolate the region containing the PVN, which was immediately snap-frozen on dry ice and stored at −80 °C. Frozen punches were Dounce-homogenized on ice in nuclei homogenization buffer (250 mM sucrose, 25 mM KCl, 5 mM MgCl_2_, 10 mM Tris–HCl pH 8.0, 1 mM DTT, 0.1% Triton X-100, Promega RNasin Plus RNase inhibitor 40 U/μL, Invitrogen SUPERase-In 20 U/μL, 1*x* Roche cOmplete EDTA-free protease inhibitor, Hoechst 33,342) and passed through a 35-μm filter. Nuclei were sorted on a BD Biosciences FACSAria II system equipped with a 70-μm nozzle directly into RLT Plus buffer (Qiagen) supplemented with β-mercaptoethanol. Single nuclei were gated by FSC/SSC and Hoechst fluorescence to remove debris and aggregates. From the Hoechst-positive population, mScarlet-positive and mScarlet-negative nuclei were collected separately. Nuclei were then used for RNA extraction and qPCR as described in that section.

### 4.8 Immunofluorescence and imaging

Mice were deeply anesthetized and perfused transcardially with ice-cold PBS followed by 4% paraformaldehyde (PFA; Electron Microscopy Sciences, 15,714). After extraction, brains were post-fixed in 4% PFA overnight at 4 °C, cryoprotected in 30% sucrose solution, embedded in M1 matrix (Epredia, 1310) and stored at −80 °C. Coronal sections (35 μm) containing the hypothalamus were cut on a cryostat and stored in PBS at 4 °C until staining. Mice for Supplementary Fig. 1 were perfused at 20 weeks, for Fig. 3 at 6 weeks, and for Fig. 4 at 20 weeks.

For immunostaining, free-floating sections were rinsed in PBS and incubated for 1 h at room temperature in blocking solution containing 10% goat serum (Jackson Immunoresearch, 005-000-121), 0.3% Triton X-100 (Sigma, *X*100-500 ML), and 90% antibody buffer (0.75% Tris base [Thermo Fisher Scientific, BP152], 1.8 % l-Lysine [Alfa Aesar, J62225], 1.125% NaCl [MilliporeSigma, S5886], 1% BSA [Thermo Fisher Scientific, BP1600], and 0.04% sodium azide [Millipore Sigma]; pH adjusted to 7.4) and then incubated overnight at 4 °C with primary antibodies diluted in blocking solution (rabbit anti-CEP43 [Proteintech, 11343-1-AP, 1:750], mouse anti-RFP [Rockland, 200-301-379, 1:250], and chicken anti-ADCY3 [antibodies.com, A104341, 1:500], mouse anti-FLAG M2 [Sigma, F1804, 1:500]). Following primary incubation, sections were washed and incubated in blocking solution with appropriate Alexa Fluor-conjugated secondary antibodies (goat anti-rabbit IgY (H + L) Alexa Fluor 405 [abcam, ab175652, 1:750], GFP Booster Alexa Fluor 488 [Proteintech, gb2AF488, 1:500], goat anti-mouse IgG2a (γ2a) Alexa Fluor 546 [Invitrogen, A21133, 1:500], goat anti-chicken IgY (H + L) Alexa Fluor 633 [Invitrogen, A21103,1:500], goat anti-mouse IgG1 (γ1)Alexa Fluor 633 [Invitrogen, A21126, 1:500]) for 1 h at room temperature. Sections were then rinsed in PBS, counterstained with Hoechst 33,342 (Invitrogen, H3570), if applicable, and mounted using ProLong Diamond Antifade Mountant (Invitrogen, P36970).

Images were collected on a Nikon CSU-W1/SoRa spinning-disk confocal microscope equipped with dual Hamamatsu ORCA-FusionBT cameras. PVN images at 20 × were acquired as single-plane fields (746 μm × 746 μm). High-resolution 60 × z-stacks were collected with a 0.2 μm step size (field size: 249.6 μm × 249.6 μm). Image insets reflect maximum-intensity projections generated in Fiji with identical imaging parameters applied to all samples within each experiment.

### 4.9 Cilia quantification

Quantification of primary cilia was performed following the methodology described in Yue et al. [91], with minor adaptations to accommodate differences in imaging channels. For Fig. 3, a new convolutional neural network–based segmentation model in NIS-Elements (Nikon) was trained using the segment. ai module, following the same training procedures described in the published method but using different antibody–channel combinations. Downstream measurements of fluorescence intensity were performed using the same overall workflow as described in Yue et al. For the dataset in Fig. 4, cilia were segmented manually in FIJI on maximum-intensity projections, and fluorescence measurements were obtained from sum-intensity projections with local background subtraction using a dilated background region, consistent with the analytical approach described in the reference. Manual segmentation was used for this dataset because attempts to segment the images automatically yielded poor performance due to differences in image quality and channel combinations.

### 4.10 Statistical analysis

Sample sizes were chosen based on expected effect sizes from prior publications and from preliminary experiments performed in this study. Data distribution was assumed to be normal, although this was not formally tested. All statistical tests used for each dataset are reported in the corresponding figure legends. Data analyses were performed using GraphPad Prism version 10.4.1 (GraphPad Software), and a significance threshold of P ≤ 0.05 was applied throughout. For analyses involving multiple measurements per biological replicate, data were summarized and compared using the SuperPlots approach [92] and the SuperPlotsOfData online tool (https://huygens.science.uva.nl/SuperPlotsOfData/). All data are presented as mean ± SEM unless otherwise indicated.

## CRediT authorship contribution statement

**Abbey Diener Blake:** Writing – review & editing, Writing – original draft, Supervision, Methodology, Investigation, Conceptualization. **Zhixuan Zhang:** Methodology, Investigation, Conceptualization. **Coralie Alexandre:** Methodology, Investigation. **Anne Gorayeb:** Investigation. **Mrinaalini Jain:** Investigation. **Qi Liu:** Investigation. **Rithu Pattali:** Investigation. **Alicja Bednarska:** Investigation. **Parleen Kaur Pandher:** Investigation. **Sumei Zhang:** Investigation. **Xinyu Yue:** Methodology, Investigation, Conceptualization. **James Nuñez:** Writing – review & editing, Supervision, Funding acquisition, Conceptualization. **Christian Vaisse:** Writing – review & editing, Supervision, Investigation, Funding acquisition, Conceptualization.

## Ethics declaration

This study was conducted in accordance with the ARRIVE (Animal Research: Reporting of In Vivo Experiments) guidelines. This study was approved by the UCSF IACUC. (Approval No. AN201856)

## Declaration of generative AI and AI-assisted technologies in the manuscript preparation process

During the preparation of this work the authors used ChatGPT (OpenAI) to assist with organization of text and refinement of clarity. After using this tool/service, the author(s) reviewed and edited the content as needed and take full responsibility for the content of the published article.

## Declaration of competing interests

The authors declare that they have no known competing financial interests or personal relationships that could have appeared to influence the work reported in this paper.

## Data Availability

Data will be made available on request.
